# A continental-wide molecular approach unraveling mtDNA diversity and geographic distribution of the Neotropical genus *Hoplias*

**DOI:** 10.1371/journal.pone.0202024

**Published:** 2018-08-13

**Authors:** Yamila P. Cardoso, Juan J. Rosso, Ezequiel Mabragaña, Mariano González-Castro, Matías Delpiani, Esteban Avigliano, Sergio Bogan, Raphael Covain, Nahuel F. Schenone, Juan M. Díaz de Astarloa

**Affiliations:** 1 Laboratorio de Sistemática y Biología Evolutiva, Facultad de Ciencias Naturales y Museo, Universidad Nacional de La Plata, La Plata, Buenos Aires, Argentina; 2 Consejo Nacional de Investigaciones Científicas y Técnicas, Buenos Aires, Argentina; 3 Grupo de Biotaxonomía Morfológica y Molecular de Peces, Instituto de Investigaciones Marinas y Costeras, Universidad Nacional de Mar del Plata, Mar del Plata, Argentina; 4 Fundación Bosques Nativos Argentinos para la Biodiversidad, Buenos Aires, Argentina; 5 Instituto de Investigaciones en Producción Animal, Facultad de Ciencias Veterinarias, Universidad de Buenos Aires, Buenos Aires, Argentina; 6 Fundación de Historia Natural “Félix de Azara”, Departamento de Ciencias Naturales y Antropología, Universidad Maimónides, Buenos Aires, Argentina; 7 Department of Herpetology and Ichthyology, Museum of Natural History, Geneva, Switzerland; 8 Centro de Investigaciones Antonia Ramos, Villa Bonita, Campo Ramón, Misiones, Argentina; National Cheng Kung University, TAIWAN

## Abstract

With an estimate of around 9,000 species, the Neotropical region hosts the greatest diversity of freshwater fishes of the world. Genetic surveys have the potential to unravel isolated and unique lineages and may result in the identification of undescribed species, accelerating the cataloguing of extant biodiversity. In this paper, molecular diversity within the valuable and widespread Neotropical genus *Hoplias* was assessed by means of DNA Barcoding. The geographic coverage spanned 40 degrees of latitude from French Guiana to Argentina. Our analyses revealed 22 mitochondrial lineages fully supported by means of Barcode Index Number, Automatic Barcode Gap Discovery and phylogenetic analyses. This mtDNA survey revealed the existence of 15 fully supported mitochondrial lineages within the once considered to be the continentally distributed *H*. *malabaricus*. Only four of them are currently described as valid species however, leaving 11 mitochondrial lineages currently “masked” within this species complex. Mean genetic divergence was 13.1%. Barcoding gap analysis discriminated 20 out of the 22 lineages tested. Phylogenetic analyses showed that all taxonomically recognized species form monophyletic groups. *Hoplias malabaricus sensu stricto* clustered within a large clade, excluding the representatives of the La Plata River Basin. In the *H*. *lacerdae* group, all species but *H*. *curupira* showed a cohesive match between taxonomic and molecular identification. Two different genetic lineages were recovered for *H*. *aimara*. Given the unexpected hidden mitochondrial diversity within *H*. *malabaricus*, the COI sequence composition of specimens from Suriname (the type locality), identified as *H*. *malabaricus sensu stricto*, is of major importance.

## Introduction

The Neotropical region hosts the greatest diversity of freshwater fishes in the world [1]. Even after centuries of research and the on-going description of new species, thousands of species remain unknown to science [2,3]. Taxonomy has long been the primary source of information for our understanding of species richness. Biodiversity estimates based solely on morphology can be handicapped by often-pervasive underestimates of cryptic taxa *sensu* Mayr [4]. Cryptic species undergoing radiation without morphological changes are not detected by traditional taxonomy, and the existence of genetically different entities within a supposed unique nominal taxon has been reported many times during the last decades [5]. In a review, Teletchea [6] provided an extensive analysis of available PCR-methods for aiding in species identification, including DNA Barcoding. DNA Barcoding has proved to be an important tool to detect cryptic diversity [7–11] and to flag potential undescribed taxa [12]. Indeed, some authors have already detected cryptic species and described new ones using an integrative approach combining DNA Barcoding and traditional taxonomy [13–20]. Application of DNA barcoding has also effectively facilitated species identification of unknown samples for conservation purposes [21].

During the last few decades, genetic studies have shown that several emblematic species from the freshwater fish fauna of South America include unanticipated levels of cryptic diversity [22–24]. Among them, *Hoplias malabaricus* (Bloch, 1794) has been intensively studied by means of karyological [25–30] and molecular [31,32] approaches. Since the foundational studies of Bertollo et al. [33], *H*. *malabaricus* has been considered a well-populated species complex, with eight recognized karyomorphs that vary in diploid number, chromosome morphology and the presence of sex chromosome systems [25,28,34]. However, it has already been shown that a single karyomorph of *H*. *malabaricus* may harbour more than one species [32]. The diversity among different populations of the same karyomorph usually is evaluated by means of in situ hybridization techniques [35,36]. Indeed, karyomorph A has shown substantial differences among allopatric populations as detected by molecular chromosome markers [29,37,38]. Molecular data also showed high divergence between populations of *H*. *malabaricus* from different basins of Brazilian Atlantic drainages [39]. Moreover, DNA Barcoding has shown that *H*. *malabaricus* from the southernmost extreme of the species´ distribution range represents a different lineage to counterparts from other basins in South America [24,32]. All these results clearly demonstrate the existence of a strong geographic structure in karyomorphs and mitochondrial lineages of this species complex.

*Hoplias malabaricus* is one of the 14 valid nominal species within the genus *Hoplias* [40,41], regionally known as thrairas. Species of *Hoplias* may be classified into three different groups using morphological characters [42]: the monotypic *H*. *aimara* (Valenciennes 1847) *group* [43], the *H*. *lacerdae* group (*H*. *lacerdae* Miranda Ribeiro 1908, *H*. *australis* Oyakawa and Mattox 2009, *H*. *brasiliensis* (Spix & Agassiz 1829), *H*. *intermedius* (Günther 1864) and *H*. *curupira* Oyakawa and Mattox 2009) and the *H*. *malabaricus* group (*H*. *malabaricus*, *H*. *microlepis* (Günther 1864), *H*. *teres* (Valenciennes 1847), *H*. *misionera* Rosso, Mabragaña, González-Castro, Delpiani, Avigliano, Schenone and Díaz de Astarloa 2016, *H*. *argentinensis* Rosso, González-Castro, Bogan, Cardoso, Mabragaña, Delpiani and Díaz de Astarloa 2018 and *H*. *mbigua* Azpelicueta, Benítez, Aichino and Mendez 2015. *Hoplias patana* (Valenciennes 1847) and *H*. *microcephalus* (Agassiz 1829) are valid species without a formally recognized group. Recent ichthyological surveys have yielded important findings regarding the diversity of *Hoplias* in South America, and five new species have been described [44–47].

These studies show that the number of species in the genus *Hoplias* and the genetically distinct groups within the *H*. *malabaricus* species complex inhabiting the freshwater ecosystems of the Neotropical region remain unknown. Our knowledge of the diversity within the genus *Hoplias* would be greatly improved by a molecular introspective analysis using the DNA Barcoding. Accurate identification of thrairas is essential for freshwater biodiversity research, because of the economic value of these species in subsistence and commercial fisheries throughout South America [48–51]. Some species of *Hoplias* can attain considerable size; *H*. *lacerdae* and *H*. *aimara* are among the giants of the group, reaching up to one meter [44], making them a target species for game fishing. Furthermore, the thrairas play an essential role in freshwater ecosystems and as top predators [52], these large carnivorous species may control the structure and abundance of fish communities.

In this paper we tested the molecular diversity within the genus *Hoplias* in South America using DNA Barcoding. Our main goal was to explore the *H*. *malabaricus* species complex in order to demonstrate molecular discrimination between recently described species and the remaining undescribed operational taxonomic units. We therefore included molecular data from the type locality of *H*. *malabaricus* from Suriname [53,54] as a benchmark for the *H*. *malabaricus* collected in other drainages of the continent. Molecular diversity within the *H*. *lacerdae* group, and the monotypic *H*. *aimara* was also explored.

## Materials and methods

### Study area and field sampling

Fishing effort was concentrated in the La Plata River and Guyana Shield basins covering a geographic range that surpasses 40 latitudinal degrees (5.55 N to 35.6 S; 40.51 E to 76.41 W). In Argentina, major sub-catchments sampled were the middle and lower Uruguay and Paraná, lower Paraguay, La Plata, Salí-Dulce, Iguazú, Pilcomayo and Bermejo rivers. In Suriname: the Nickerie, Saramacca, Corantijne, Suriname, Marowijne, Commewijne and Coppename rivers. In French Guiana: the Approuague, Kaw, Kourou, Sinnamary and Organabo rivers. In Peru: the Huallaga and Ucayali rivers. Samples from elsewhere in South America were gathered either by donation or from public databases. Fishing was mostly by seine and hand netting in order to minimize fish stress upon capture. When habitat conditions (water depth, water velocity) preclude using these methods, gill or trammel netting was used. After deployment, nets were regularly checked for freshly entangled fishes thereby minimizing their stress. The specimens collected were identified using the original descriptions and updated taxonomical literature [43–46,55]. Morphological vouchers were deposited in the fish collections of the Fundación de Historia Natural “Felix de Azara”, Buenos Aires (CFA-IC), the Museum d’ Histoire Naturelle, Geneva (MHNG), and the Instituto de Investigaciones Marinas y Costeras, Mar del Plata (IIMyC-UNMDP).

### Ethical statement

The species sampled are not protected under wildlife conservation laws (local restrictions, IUCN or CITES listed species). No experimental activities were conducted on live specimens in this study. After specimens were euthanized (see Methods below), a small portion of tissue from each fish was excised and preserved in 95% ethanol for genetic studies. Vouchers specimens were fixed in 10% formaldehyde, transferred to 4% formaldehyde before being shipped to the ichthyological collections for positive identification and permanent preservation in 70% ethanol. Fish were collected with the permission of the local authorities in Argentina, Peru, French Guiana and Suriname. Collection permits in Argentina were granted by Ministerio de Ecología y Recursos Naturales Renovables de Misiones (Disp. 013/16); Ministerio de Producción y Ambiente de Formosa (N° 2577/12); Dirección Natural de Recursos Naturales de Entre Ríos (Hab. Cient. 2011–2012); Secretaría de Medio Ambiente y Desarrollo Sustentable de Santa Fe (Res. 081/2015); Ministerio de Asuntos Agrarios de Buenos Aires (Res. 355/10); Dirección de Fauna y Áreas Naturales Protegidas de Chaco (Cons. Aut. 2012); Dirección de Flora, Fauna Silvestre y Suelos de Tucumán (Res. 223/15); Secretaría de Medio Ambiente y Desarrollo Sustentable de Salta (Res. 091/05) and Dirección General de Bosques y Fauna de Santiago del Estero (Ref. 17461/2015). In French Guiana and Suriname, specimens were collected and exported with appropriate permits: Préfecture de la Région Guyane, Arrété 03/17/PN/EN to collect in the Réserve Naturelle des Nouragues; Ministry of Agriculture, Animal Husbandry and Fisheries to export fishes from Suriname. Material obtained from the Parc Amazonien de Guyana was collected under the direct supervision of PAG authorities. When collecting occurred in non-protected areas of French Guiana, sampled specimens were declared to the French DEAL (French environmental protection ministry) before export. In Peru, field collection was performed under the bilateral research project between the Universidad Nacional Mayor de San Marcos and the Museum of Natural History from Geneva. Our Institutions do not possess formal Committees regarding the animal welfare and sampling protocols. Nevertheless, being aware about the importance of careful conduct in all procedures involving live fish, all work was conducted in accordance with relevant national and international guidelines. In Peru, French Guiana and Suriname, sampling protocols and fish handling conforms to legal requirements (Directive 2010/63/EU of the European Parliament and of the Council on the protection of animals used for scientific purposes), the Swiss ordinance OPAn 455.1 of OSAV, and recommendations and regulations of DETA-DGNP (permit number 20160422/01 AS). Accordingly, fish were anesthetized and killed using water containing a lethal dose of eugenol (clove oil). In Argentina, fish handling during sampling was performed following guidelines of the ethical committee of the Consejo Nacional de Investigaciones Científicas y Técnicas (CONICET) and the UFAW Handbook on the Care and Management of Laboratory Animals (http://www.ufaw.org.uk). Collection permits in Argentina are granted without a formal request concerning the protocol used for the humane killing of fish. Notwithstanding, we opted to kill the fish with an overdose of benzocaine, as recommended by the New South Wales Fisheries Animal Care and Ethics Committee [56].

### DNA extraction and PCR

DNA was extracted and amplified at the Laboratorio de Sistemática y Biología Evolutiva at La Plata, the MHNG and at the International Barcode of Life reference Laboratory of CONICET, located in the IIMyC-UNMDP. DNA was extracted using peqGOLD Tissue DNA Mini Kit (PeqLab) and highly automated protocols established at the CCDB [57]. The “barcode” region of the mitochondrial cytochrome c oxidase I gene (COI) was amplified by polymerase chain reaction (PCR) using universal primer cocktails for fish [58]. Standard PCR reactions were carried out in 12.5 μL total volume, containing about 20 ng of DNA template, 6.25 μL of 10% trehalose, 2 μL of ultrapure water, 1.25 μL of 10X PCR buffer (200 mMTris-HCl pH 8.4, 500 mMKCl), 0.625 μL MgCl2 (50 mM), 0.125 μL of each primer (0.01 mM), 0.0625 μL of each dNTP (10 mM), 0.060 μL of Platinum Taq Polymerase (Invitrogen). The following PCR cycling conditions were employed: 2 min at 95°C; 35 cycles of 0.5 min at 94°C, 0.5 min at 52°C, and 1 min at 72°C; 10 min at 72°C. PCR products of the query dataset were visualized in a 1% agarose gel. The sequencing reaction program consisted of an initial step of 2 min at 96°C and 35 cycles of 30 s at 96°C, 15 s at 55°C and 4 min at 60°C. Bidirectional sequencing was performed by the company MAGROGEN (Korea) and the Canadian Centre for DNA Barcoding (CCDB) in Ontario, Canada. All sequences were deposited in the Barcode of Life Data System [59] under the project named "Hoplias of South America” (HPRB) and also in GenBank (MG699453-MG699576).

### Molecular data analysis

All tissues derived from field sampling (115) and nine additional tissues samples obtained by donation from colleagues in Bolivia and Brazil were subjected to extraction, amplification and sequencing. Additionally, 101 sequences were obtained from GenBank and 119 from BOLD. Altogether, a set of 344 sequences of the genus *Hoplias* was included in the molecular analyses. Four additional sequences were used as outgroups, resulting in a final dataset of 348 sequences (S1 Table). The sequences were edited and aligned in BioEdit 7.0.9.0. [60]. Aligned sequences were subjected to three different analyses.

#### Diversity and distribution

The Barcode Index Number (BIN) was assigned for all sequences stored in BOLD. BIN analysis clusters barcode sequences to create Operational Taxonomic Units (OTUs) that closely reflect species groupings [61]. As such, the BIN is useful for estimating the number of species directly from the barcode records irrespective of the taxonomic diagnosis. The minimum Nearest Neighbor distance among BINs reported in BOLD for the whole dataset of the genus *Hoplias* was 1.12. Using this value as a threshold, sequences stored in GenBank were assigned to a BIN only when the percentage of similarity with a sequence with known BIN was 98.88 or higher. Other private BINs of *Hoplias* stored in BOLD were treated by analysing the Nearest Neighbor data of each BIN. The rationale behind the BIN approach was to test for hidden genetic diversity within each valid species of *Hoplias* incorporated in this study. In addition to the BIN analysis, we also explored species limits using the Automatic Barcode Gap Discovery method (ABGD) [62]. This method automatically finds the distance at which a barcode gap occurs and sorts the sequences into putative species based on this distance. Therefore, as in BIN analysis, it is applicable as an independent tool without an a priori species hypothesis, and it provides insight into whether the taxonomic identification based on morphological features has any genetic support. The ABGD was initially run with the default settings (*P* min = 0.001, *P* max = 0.1, steps = 10, *X* relative gap width = 1.5, Nb bins = 20) and K2P distance. Using a variable range of *P* max, Pulliandre et al. [62] found that at a *P* max = 0.01, groups detected by the ABGD closely matched the number of species in the original data sets. Therefore, to maximize concordance between genetic and taxonomic grouping, after running the model with the default parameters, the *P* max was set at 0.01. In the ABGD, the barcode gap is chosen as the first local maximum slope (of ranked distances) occurring after a threshold termed *dist*_*limit*_ (estimated from the *P* value given by the user) and *X* times larger than any gap in the prior intraspecific divergence [62]. In consequence of the “any” condition, we must expect that the gap will always be larger than *X* times *P*. The BIN analysis showed that the minimum distance between two mitochondrial lineages in the genus *Hoplias* was slightly over 1%. As the *P* max was set at 0.01 the *X* value was set to 1 to resemble the minimum interspecific distance (*X* (1) times *P* (0.01) = 1%) detected by the BIN analysis.

To gain further consensus among different methodologies in delimitations of OTUs, we finally explored whether groups identified by the BIN and ABGD approaches were supported by reciprocal monophyly in a Bayesian phylogenetic analysis. In this scenario, those sequences or group of sequences supported by the three methodologies are considered as a “full” OTU, whereas any other condition is interpreted as a “partial” OTU.

#### Genetic divergence

An analysis of genetic divergence was conducted in two ways. Firstly, we calculated the within-BIN distance summaries in BOLD (p-distance) since this platform contains the largest data set for each particular BIN (including private sequences to which we did not have access). Secondly, genetic distance among BINs was estimated based on our data set because BOLD only estimates divergence among species. The Tamura-Nei model (TN93) was chosen as the best nucleotide substitution model under the Bayesian Information Criterion and therefore this was used to estimate genetic divergences among BINs using Mega.7 [63]. The package ‘Vegan’ [64] was used to perform a multidimensional scaling analysis (MDS) to obtain a graphic representation of genetic distances among BINs in R [65]. The species discrimination power of DNA barcoding was analysed by plotting the maximum intra-BIN distance of each OTU in axis X against this value subtracted from the minimum distance to the nearest neighbour in axis Y. Negative values for axis Y show no resolution in barcode-gap.

#### Phylogenetic analyses

Two phylogenetic analyses were conducted disregarding BIN assignment. Firstly, Neighbor-Joining (NJ) analysis was performed using the TN93 model in MEGA.7. Confidence values for the edges of the NJ tree were computed by bootstrapping [66], with 1000 replications. Secondly, Bayesian inference (BI) analysis was conducted in MrBayes 3.2.2 [67,68] on CIPRES Science Gateway computing cluster [69]. Four chains were run simultaneously (three heated, one cold) for 30 million generations, with tree space sampled every 500th generation. After a graphical analysis of the evolution of the likelihood scores, the first 25% of generations were discarded as burn-in. All run parameters through the generations, as well as data convergence, were examined using the software Tracer 1.5 [70], and only runs with an ESS > 200 were accepted. The remaining trees were used to calculate the consensus tree using the “sumt” command in MrBayes. This command summarizes the statistics for the taxon bipartitions and generates a tree with posterior clade probability values and a phylogram using branch lengths data.

## Results

**Fig 1:**
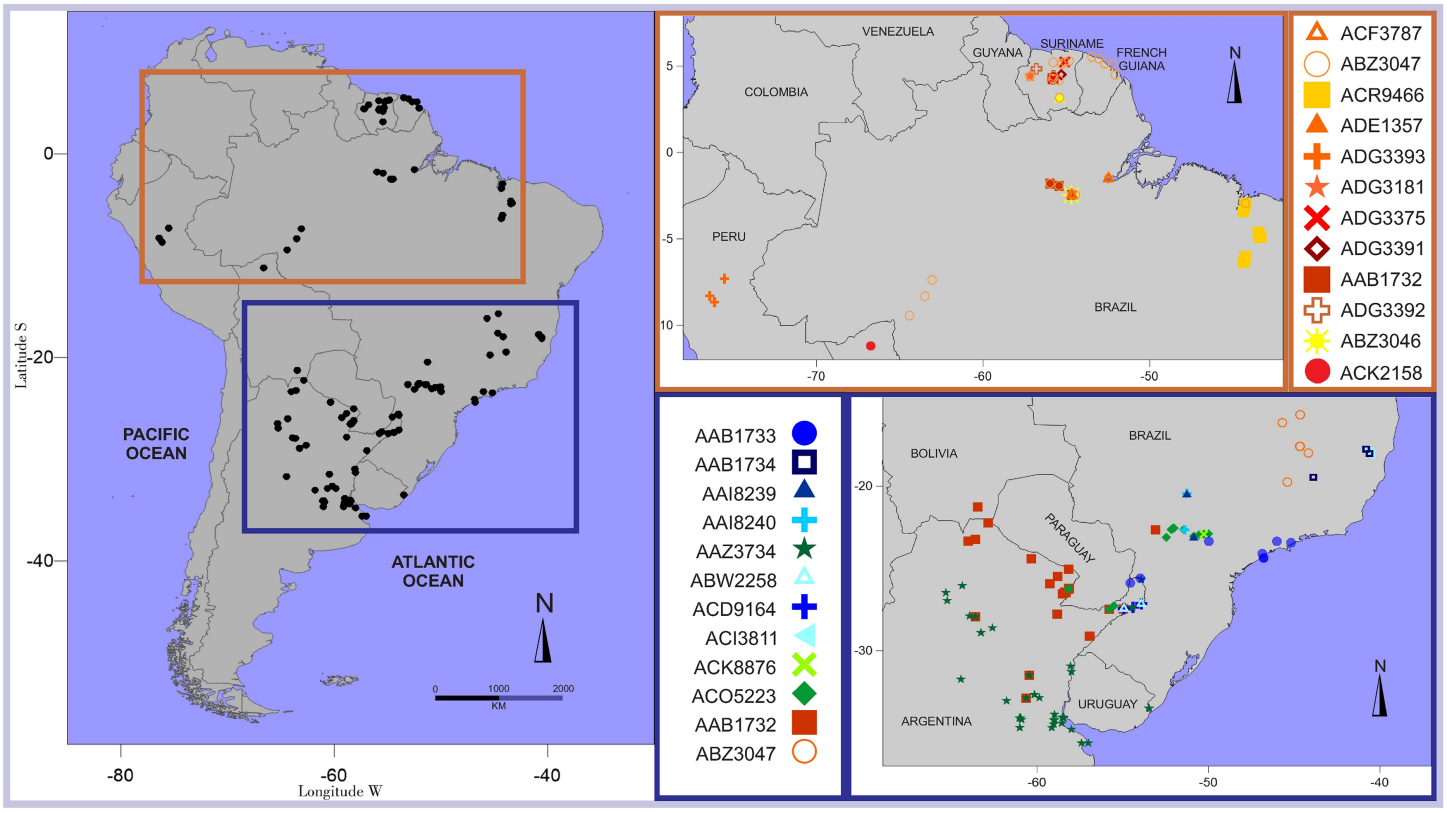
Map of the study region showing geographic distribution of different BINs.

A total of 124 fishes from 71 different localities were collected in a number of different river basins in Argentina, Brazil, Bolivia, Peru, French Guiana and Suriname (S1 Table). Our geographic coverage spanned more than 40 latitude degrees (Fig 1), ranging from 5.54° N and 53.46° W in French Guiana to 35.60° S and 57.41° W in Buenos Aires Province, Argentina.

Our amplicons for the 5´ region of the mitochondrial COI gene averaged 638 bp. No stop codons, insertions or deletions were found in any of the amplified sequences. Average nucleotide composition was 30% (T), 28.4% (C), 23.2% (A) and 18.4% (G). Among 73 analysed specimens of *Hoplias* from La Plata River Basin, 61 specimens were identified as belonging to the *H*. *malabaricus* group [*H*. *misionera* (n = 38), *H*. *mbigua* (n = 5) and *H*. *argentinensis* (n = 18)] and 12 to the *H*. *lacerdae* group [*H*. *lacerdae* (n = 5) and *H*. *australis* (n = 7)]. Specimens from rivers of Suriname and French Guiana were identified as *H*. *aimara* (n = 4), *H*. *curupira* (n = 9) and *H*. *malabaricus sensu stricto* (n = 25). Samples from the Amazon Basin in Peru (n = 4) and Bolivia (n = 1) were identified as *H*. cf. *malabaricus*.

### Diversity and distribution

Overall, 25 BINs were recovered for the genus *Hoplias* (Table 1). Two of the recovered BINs are private data and were detected by means of the Nearest Neighbor Analysis (NN) and by the Identification System of BOLD using GenBank sequences. Only three sequences from Genbank (JX112674/ JX112679/ JX112687) could not be assigned to a known BIN. A total of 16 different BINs were revealed within the *H*. *malabaricus* species complex. The BINs ACO5223, AAZ3734 and AAB1732 represent the recently described species *H*. *mbigua*, *H*. *argentinensis* and *H*. *misionera*, respectively. All sequences from Suriname and French Guiana, assigned as *H*. *malabaricus sensu stricto*, received the BIN ABZ3047.

**Table 1:** Distance summary for BINs available at BOLD of *H*. *malabaricus*, *H*. *aimara* and *H*. *lacerdae* groups.

*Hoplias malabaricus* group								
BIN	Reference	Basin	Current Taxonomy	N (public)	Max	Mean	NN	Distance to NN
AAB1732	[31,32,46,71]	Amazon, La Plata	*Hoplias misionera*	64 (24)	3.54	0.62	ABZ3047	5.3
ACO5223	[46]	La Plata	*Hoplias mbigua*	28 (25)	0.67	0.12	AAI8239	1.13
AAB1733	[72]	La Plata	*Hoplias malabaricus*	22 (20)	0.64	0.19	AAY4779	6.17
AAI8239	[31,73]	La Plata	*Hoplias intermedius* (mis ID)	2 (2)	0	0	ACO5223	1.13
AAI8240	[31]	La Plata	*Hoplias intermedius* (mis ID)	6 (6)	0.33	0.14	AAB1731	2.35
AAY4779#	Private		*Hoplias malabaricus*	7(0)	0.7	0.29		
AAZ3734	[24,71]	La Plata, Patos-Mirim	*Hoplias argentinensis*	93 (27)	1.8	0.61	ABZ3047	5.54
ABZ3046*	[32]	Amazon	*Hoplias malabaricus*	2 (0)	0.15	0.15	ACF3787	1.89
ABZ3047	[23,32]	Amazon, Guiana Shield, Itapecuru, Sao Francisco	*Hoplias malabaricus*	97 (16)	3.71	1.67	AAB1731	1.44
AAB1731	Released by BOLD	Amazon	*Hoplias malabaricus*	10 (4)	1.75	0.93	ACF3787	1.42
ACR9466	Released by BOLD	Itapecuru	*Hoplias malabaricus*	52 (46)	0.63	0.1	ABZ3047	1.76
ACF3787*	Marques et al. 2013	Amazon	*Hoplias malabaricus*	11(0)	0.61	0.14	AAB1731	1.42
ACI3811	Released by BOLD	Mucuri	*Hoplias malabaricus*	9 (5)	1.01	0.35	AAY4779	5.47
ACK2158	Released by BOLD	Amazon	*Hoplias malabaricus*	2(1)	0.5	0.5	ABZ3046	4.33
ACK8876	[73]	La Plata	*Hoplias sp*.	1 (1)	N/A	N/A	ACO5223	2.51
ADG3393	this study	Amazon	*Hoplias malabaricus*	4(0)	0.8	0.4	AAB1731	5.05
***Hoplias aimara***								
ADE1357	this study	Guiana Shield	*Hoplias aimara*	7(0)	0.16	0.04	ADG3375	2.09
ADG3375	this study	Guiana Shield	*Hoplias aimara*	2(0)	0.16	0.16	AAX1177	1.12
AAX1177#	Private		*Hoplias sp*.	2(0)	0.15	0.15		
***Hoplias lacerdae* group**								
ABW2258	this study	La Plata	*Hoplias lacerdae*	5(5)	0.48	0.27	ACD9164	5.21
ACD9164	this study	La Plata	*Hoplias australis*	8(6)	0.51	0.24	ABW2258	5.46
ADG3181	this study	Guiana Shield	*Hoplias curupira*	2(0)	0	0	ADG3392	3.53
ADG3391	this study	Guiana Shield	*Hoplias curupira*	2(0)	1.61	1.61	ADG3392	2.25
ADG3392	this study	Guiana Shield	*Hoplias curupira*	5(0)	0.32	0.19	ADG3391	2.25
AAB1734	[23]	Sao Francisco, Mucuri	*Hoplias intermedius*	17 (4)	2.43	0.76	ADE1357	9.76

*: BIN recovered by means of the Identification System of BOLD when using GenBank sequences.

^#^: BIN recovered by means of the Nearest Neighbour Analysis. NN: the nearest neighbour BIN. Max: maximum intra-BIN distance. Mean: mean within-BIN distance.

Within the *H*. *lacerdae* group, six BINs were recovered. Surprisingly, three BINs were reported for specimens identified as *H*. *curupira*. *Hoplias intermedius*, *H*. *lacerdae and H*. *australis* were identified with one private BIN each. In the monotypic *H*. *aimara* group, three genetic lineages were recovered, one of them found by the Nearest Neighbor Analysis.

The analysis of the ABGD dataset using the default parameters resulted in four partitions that ranged from 121 (*P* max = 0.001) to one candidate species (*P* max = 0.005), with two partitions with 56 candidate species (*P* max = 0.002, *P* max = 0.003). However, when the minimum relative gap width was set to 1 and the *P* max to 0.01, the number of groups decreased to 26 (*P* max = 0.0046 to *P* max = 0.01) in four of ten partitions, a result that is clearly more consistent with the BIN analysis. The sequences compositions of the different BINs were fully recovered by the groups proposed by the ABGD, with the exception of ABZ3047 and ADG3391. The ABGD grouped ABZ3047 (*H*. *malabaricus sensu stricto*) with the singletones JX112679 and JX112687 (*H*. *malabaricus*) as a single group (Group 1), whereas the ADG3391 (*H*. *curupira*) was further split into two groups (Group 7 and 8 in Table 2).

**Table 2:** Identification of OTUs in the genus *Hoplias* by means of Barcode Index Number (BIN), Automatic Barcode Gap Discovery (ABGD) and reciprocal monophyly in a Bayesian and NJ analyses.

Current Taxonomy	BIN	ABGD group	Reciprocal monophyly	OTU match
*Hoplias malabaricus group*				
*Hoplias misionera*	AAB1732	17	Yes	Full
*Hoplias mbigua*	ACO5223	2	Yes	Full
*Hoplias argentinensis*	AAZ3734	13	Yes	Full
*Hoplias malabaricus*	AAB1733	4	Yes	Full
*Hoplias intermedius* (mis ID)	AAI8239	26	Yes	Full
*Hoplias intermedius* (mis ID)	AAI8240	3	Yes	Full
*Hoplias malabaricus*	AAY4779	---	---	Partial
*Hoplias malabaricus*	ABZ3046	21	Yes	Full
*Hoplias malabaricus sensu stricto*	ABZ3047	1	No	Partial
*Hoplias malabaricus*	AAB1731	14	Yes	Full
*Hoplias malabaricus*	ACR9466	11	Yes	Full
*Hoplias malabaricus*	ACF3787	18	Yes	Full
*Hoplias malabaricus*	ACI3811	15	Yes	Full
*Hoplias malabaricus*	ACK2158	25	Yes	Full
*Hoplias sp*.	ACK8876	22	Yes	Full
*Hoplias malabaricus*	ADG3393	12	Yes	Full
*Hoplias malabaricus* JX112674	JX112674	19	Yes, singleton	Full
*Hoplias malabaricus* JX112679	JX112679	1	with a clade of ABZ3047	Partial
*Hoplias malabaricus* JX112687	JX112687	20	with a clade of ABZ3047	Partial
*Hoplias aimara*				
*Hoplias aimara*	ADE1357	9	Yes	Full
*Hoplias aimara*	ADG3375	10	Yes	Full
*Hoplias sp*.	AAX1177	---	---	Partial
*Hoplias lacerdae group*				
*Hoplias lacerdae*	ABW2258	24	Yes	Full
*Hoplias australis*	ACD9164	23	Yes	Full
*Hoplias curupira*	ADG3181	6	Yes	Full
*Hoplias curupira*	ADG3391	7, 8	No	Partial
*Hoplias curupira*	ADG3392	5	Yes	Full
*Hoplias intermedius*	AAB1734	16	Yes	Full

OTU match: Full (all three approaches in agreement); Partial (at least one approach in disagreement).

Most OTUs of the *H*. *malabaricus* species complex were restricted to either the La Plata River or the Amazon drainages (Table 1, Fig 1). Conversely, the BIN AAB1732 (Group 17 in ABGD) of *H*. *misionera* presented a wider geographic distribution in South America, clustering sequences from the Lower Paraná, Middle Paraná, Paraguay and Amazon rivers. The OTU containing *H*. *malabaricus sensu stricto* (AAZ3047; Group 1 in ABGD) from Suriname and French Guiana also clustered specimens from the Lower and Upper Amazon River and the north-eastern rivers of Brazil. The La Plata River and Patos-Mirim basins shared the OTU defined by the BIN AAZ3734 and the Group 13 in ABGD, which correspond to the recently described *H*. *argentinensis*. Sequences of *H*. *curupira* and *H*. *aimara* were restricted to the Guiana Shield and *H*. *lacerdae* and *H*. *australis* to the Uruguay River drainage. Three different OTUs from the La Plata River Basin were assigned to *H*. *intermedius*, but only one of these (AAB1734; Group 16 in ABGD) contained sequences collected near the type locality (Sao Francisco River).

### Genetic divergence

The overall K2P genetic distance intra-BIN averaged 0.4%, ranging from 0% to 3.71% (Table 1). As expected, the highest intraspecific divergence was recorded in BINs with widest range distribution: *H*. *malabaricus sensu stricto* (ABZ3047) and *H*. *misionera* (AAB1732) (Fig 1). For *H*. *intermedius* (AAB1734) the maximum intraspecific divergence was 2.43%. In the remaining BINs, the intra-BIN distance did not surpass 1.8%. Within the *H*. *malabaricus* group the mean of intra-BIN distance was 0.41%, while those within the *H*. *aimara* group and the *H*. *lacerdae* group mean intra-BIN distances were 0.12% and 0.51% respectively.

**Fig 2:**
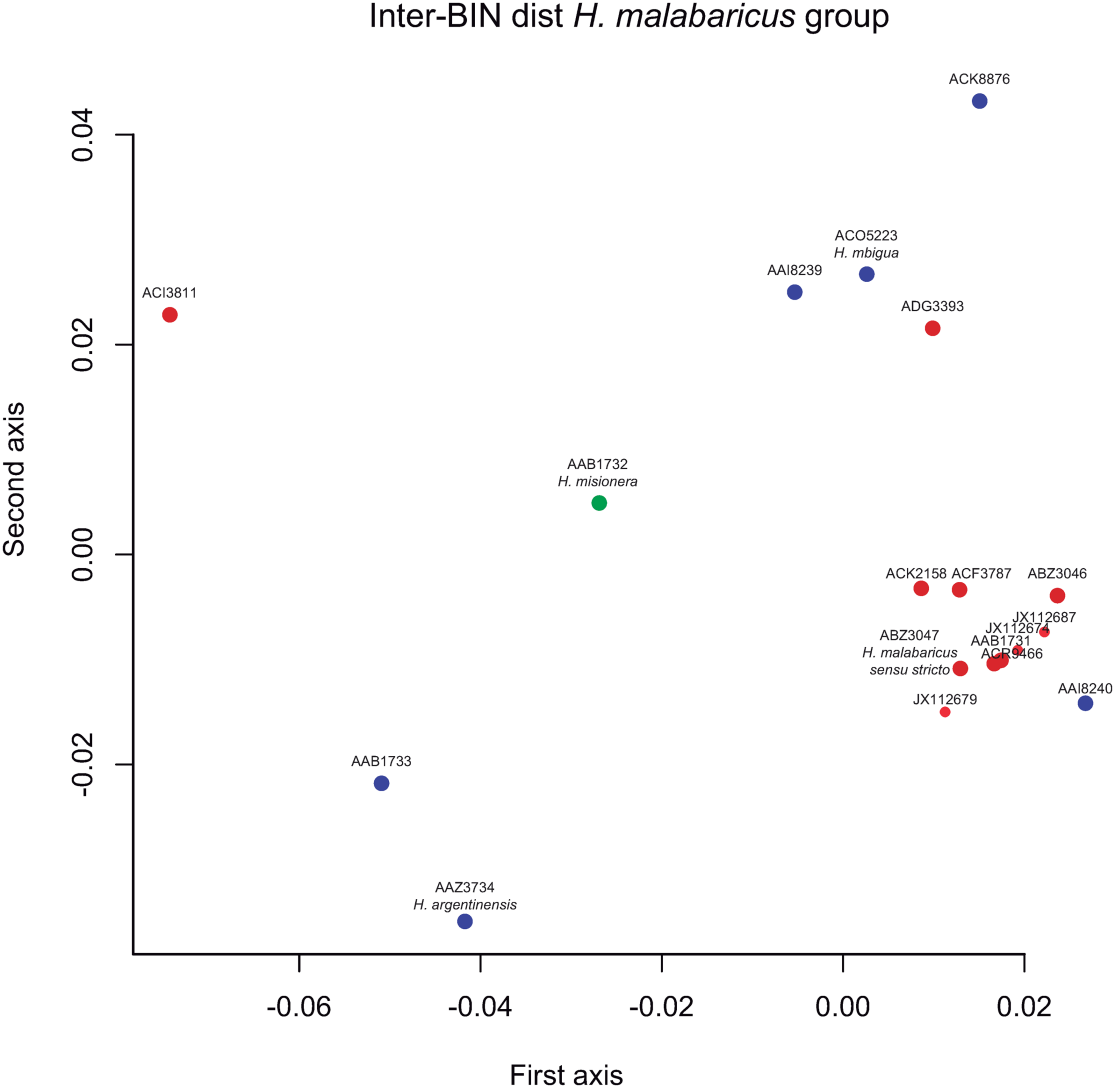
Multidimensional scaling. Multidimensional scaling analysis depicting the inter-BIN Tamura-Nei model distances matrix. Blue circles: BINs from southern drainages (La Plata Basin and Dos Patos- Mirim Lagoon); red circles: BINs from northern drainages (Amazon, Guiana Region and rivers of northeastern Brazil), Sao Francisco River and coastal rivers of Brazil; green circle: BIN present in southern and northern drainages.

The average genetic divergence among BINs (S2 Table) was large, ranging from 1.3 to 27.1% (mean = 13.1%). For the *H*. *malabaricus* species complex (Table 3) mean genetic divergence was 6.5% (with a maximum of 12.2%) and only eight of 16 BINs were separated from the Nearest Neighbor (NN) by more than 2% (Table 1). Mitochondrial lineages of fishes from the La Plata River Basin were clearly more divergent than those for most of their counterparts from elsewhere in South America which showed a more cohesive grouping (Fig 2). Within the *H*. *lacerdae* group, the inter-BIN distance was somewhat higher with a maximum of 23.8%. *Hoplias curupira* showed high intraspecific divergence and accordingly received three different BINs. Genetic divergence among BINs of *H*. *curupira* ranged from 2.5 to 4.3%. Based on our data, the genetic entities detected within *H*. *aimara* showed a divergence of 2.2% (Table 3). However, the private BIN (AAX1177) detected in BOLD showed low divergence (1.12%) with BIN ADG3375 (Table 1).

**Table 3:** Matrixes of genetic divergences.

***Hoplias aimara* group**																		
		ADG3375																
*Hoplias aimara*	ADG3375																	
*Hoplias aimara*	ADE1357	0.022																
	***Hoplias lacerdae* group**																	
		AAB1734	ABW2258	ACD9164	ADG3181	ADG3391												
*Hoplias intermedius*	AAB1734																	
*Hopias lacerdae*	ABW2258	0.127																
*Hoplias australis*	ACD9164	0.124	0.061															
*Hoplias curupira*	ADG3181	0.238	0.209	0.217														
*Hoplias curupira*	ADG3391	0.218	0.197	0.205	0.043													
*Hoplias curupira*	ADG3392	0.223	0.193	0.203	0.036	0.025												
	***Hoplias malabaricus* group**																	
		AAB1731	AAB1732	AAB1733	AAI8239	AAI8240	AAZ3734	ABZ3046	ABZ3047	ACF3787	ACI3811	ACK2158	ACK8876	ACO5223	ACR9466	ADG3393	JX112674	JX112679
*Hoplias* cf. *malabaricus*	AAB1731																	
*Hoplias misionera*	AAB1732	0.080																
*Hoplias* cf. *malabaricus*	AAB1733	0.084	0.095															
*Hoplias intermedius* (mis ID)	AAI8239	0.043	0.075	0.077														
*Hoplias intermedius* (mis ID)	AAI8240	0.030	0.090	0.087	0.048													
*Hoplias argentinensis*	AAZ3734	0.077	0.085	0.078	0.081	0.085												
*Hoplias* cf. *malabaricus*	ABZ3046	0.030	0.079	0.095	0.044	0.031	0.085											
*Hoplias malabaricus*	ABZ3047	0.028	0.077	0.088	0.049	0.044	0.077	0.040										
*Hoplias* cf. *malabaricus*	ACF3787	0.025	0.078	0.087	0.042	0.037	0.079	0.027	0.031									
*Hoplias* cf. *malabaricus*	ACI3811	0.106	0.101	0.092	0.080	0.122	0.089	0.111	0.101	0.097								
*Hoplias* cf. *malabaricus*	ACK2158	0.053	0.090	0.099	0.062	0.056	0.080	0.048	0.064	0.056	0.106							
*Hoplias* cf. *malabaricus*	ACK8876	0.061	0.095	0.107	0.032	0.067	0.102	0.062	0.067	0.061	0.109	0.077						
*Hoplias mbigua*	ACO5223	0.043	0.072	0.087	0.013	0.051	0.084	0.040	0.046	0.040	0.088	0.056	0.027					
*Hoplias* cf. *malabaricus*	ACR9466	0.034	0.088	0.093	0.058	0.050	0.084	0.046	0.032	0.037	0.103	0.065	0.072	0.054				
*Hoplias* cf. *malabaricus*	ADG3393	0.069	0.089	0.101	0.066	0.077	0.106	0.074	0.072	0.068	0.112	0.079	0.083	0.066	0.068			
*Hoplias* cf. *malabaricus*	JX112674	0.016	0.087	0.091	0.047	0.038	0.081	0.030	0.029	0.023	0.103	0.058	0.066	0.048	0.035	0.069		
*Hoplias* cf. *malabaricus*	JX112679	0.039	0.084	0.094	0.060	0.055	0.082	0.041	0.025	0.035	0.096	0.071	0.079	0.057	0.029	0.077	0.034	
*Hoplias* cf. *malabaricus*	JX112687	0.023	0.082	0.096	0.047	0.042	0.081	0.033	0.020	0.027	0.107	0.061	0.066	0.044	0.035	0.065	0.020	0.024

Matrixes of genetic divergence between BINs for different species groups of the genus *Hoplias*. Distances estimated by the Tamura-Nei model with a gamma distribution (shape parameter = 1). In the *H*. *malabaricus* group, three sequences without known BIN are also computed in the analysis.

**Fig 3:**
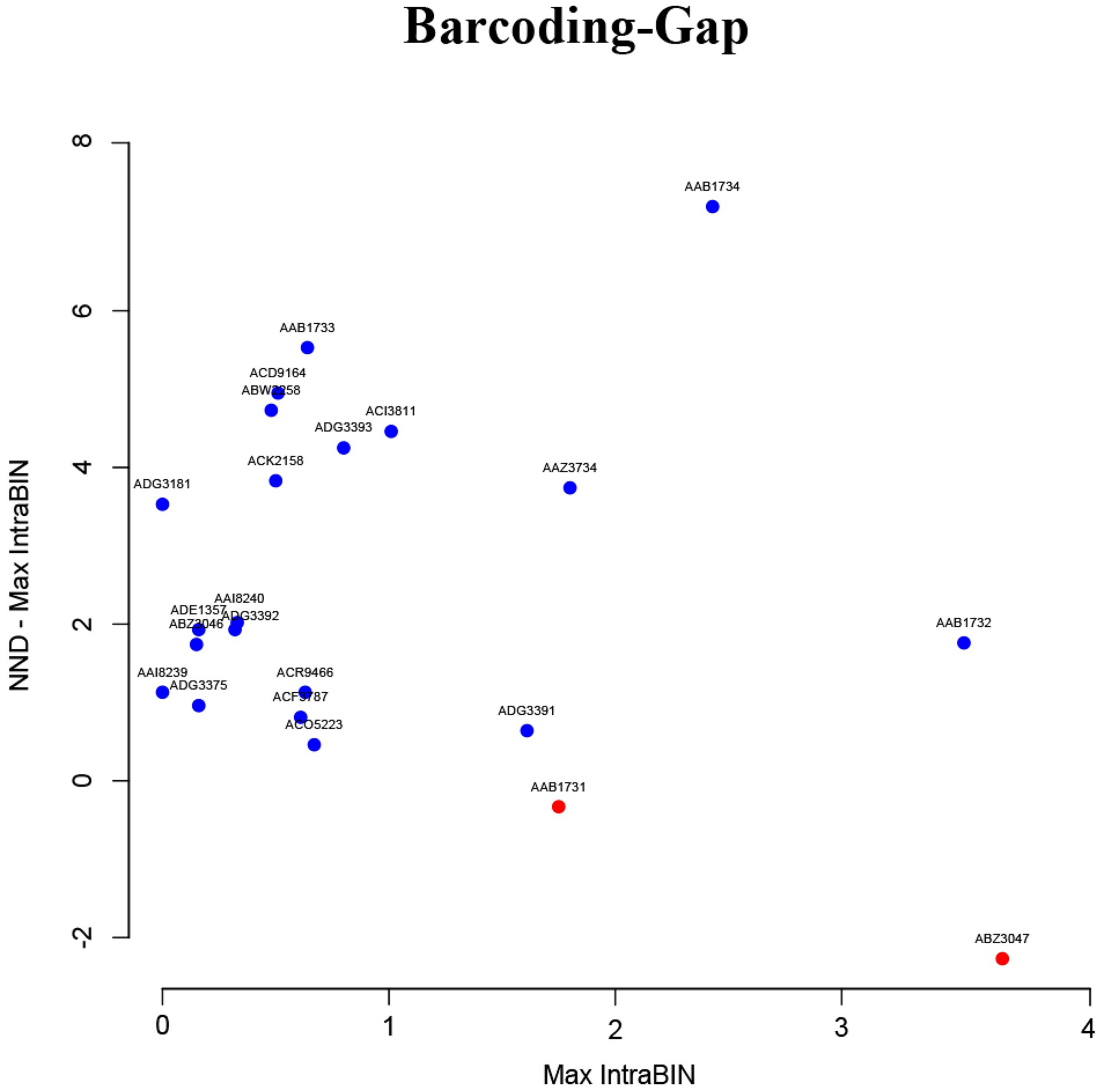
Scatterplot. Scatterplot showing BIN discrimination power of DNA-Barcode. Negatives values on axis Y show no resolution in barcode-gap (red circles). The singleton ACK8876 and those BINs recovered by the Nearest Neighbor Analysis (AAY4779, AAX1177) could not be tested (see Table 1).

Barcoding gap analysis discriminated more than 90% of BINs for the genus *Hoplias* (Fig 3). With the exception of BIN ABZ3047 and BIN AAB1731, distances of each BIN to their NN were consistently higher than the maximum intra-BIN genetic distance. The minimum distances to their NN were also calculated for the three sequences without an assigned BIN. The sequence JX112674 was 1.64% divergent from BIN AAB1731, whereas sequences JX112679 and JX112687 were 2.5% and 2% divergent from BIN ABZ3047, respectively.

### Phylogenetic analyses

**Fig 4:**
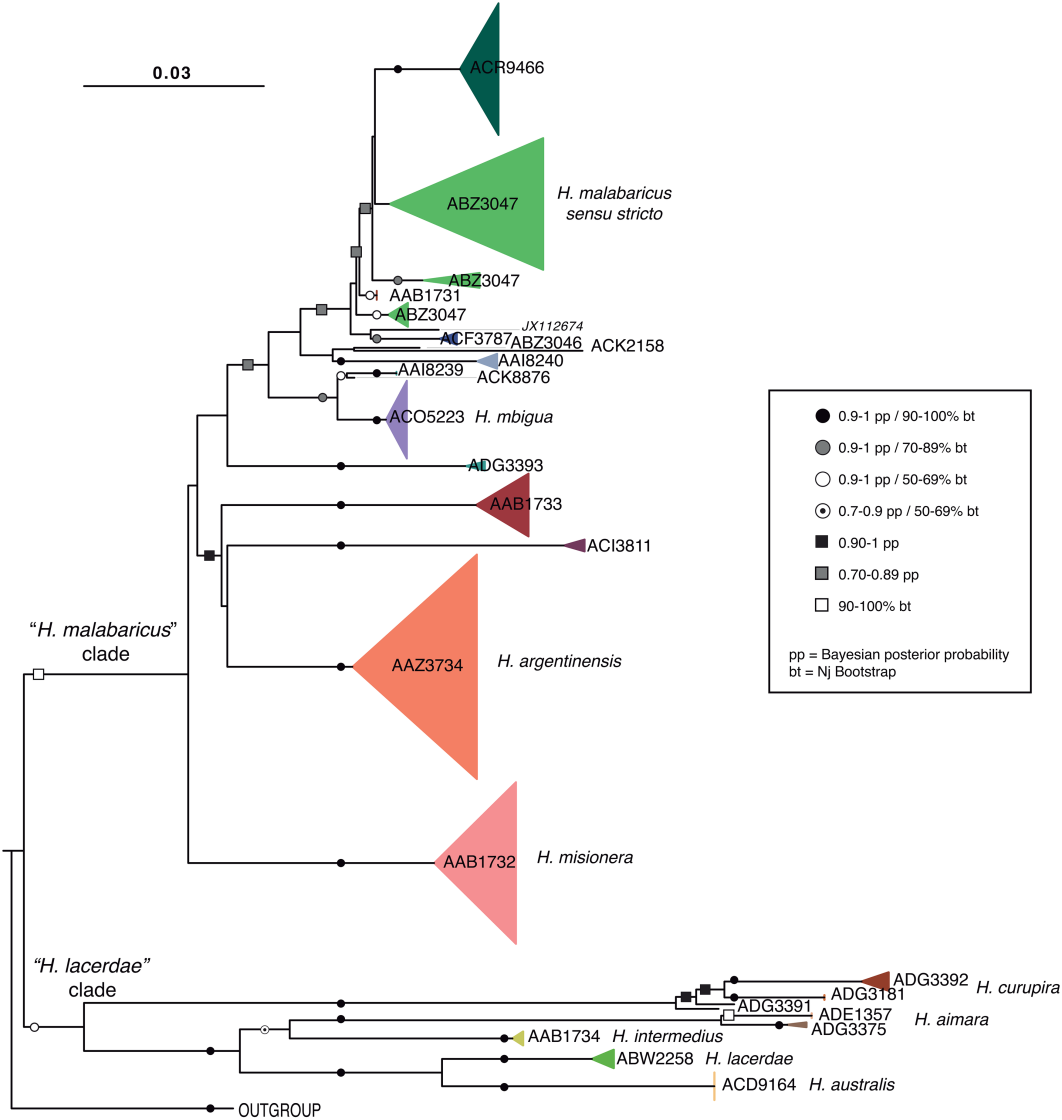
COI NJ tree. NJ tree of the COI sequences for species of *Hoplias* obtained with MEGA. Bootstrap (bt) and Bayesian posterior probability (pp) support values are indicated on nodes as: black circles (0.9–1 pp and 90–100% bt) grey circles (0.9–1 pp and 70–89% bt), white circles (0.9–1 pp and 50–69% bt), white circles with point (0.7–0.9 pp and 50–69% bt), black squares (0.9–1 pp and <50% bt), grey square (0.7–0.89 pp and <50% bt) and white squares (<0.7 pp and 90–100% bt).

Relationships among sequences were represented by NJ and BI trees. The trees show compatible topologies (Fig 4) and in both, all specimens identified as belonging to the *H*. *malabaricus* group clustered together. *Hoplias aimara* clustered within the *H*. *lacerdae* group with strong statistical support. All taxonomically recognized species were monophyletic.

Within the *H*. *malabaricus* clade, some of the internal branches relating major lineages were short and, hence, the statistical support was generally low, generating a basal polytomy in the BI tree due to the collapse of some branches supported by posterior probabilities lower than 0.5 (data not shown). Nevertheless, several strongly supported and reciprocally monophyletic lineages emerge, most of them with long branches. Moreover, each of these lineages seems to be restricted to one BIN and to a single basin. Only the BIN ABZ3047 was recovered as paraphyletic with three distinct sub-clades.

Within the *H*. *lacerdae* clade, all of the internal branches relating species were long and strongly supported. There was a clear differentiation between species, and the different BINs identities of each species nested together.

## Discussion

### Effectiveness and applications of DNA Barcoding

This study analysed the mitochondrial diversity in nine (from a total of 14) nominal valid species of the genus *Hoplias* covering a wide distribution range. It shows the potential of the DNA barcode approach to confirm field identifications, detect misidentifications (see below) and to flag up hidden mitochondrial diversity in a highly diversified species complex with taxonomic uncertainties. The BIN and ABGD analyses were able to discriminate most of the identified species. Moreover, mtDNA proved to be a powerful means of identifying genetic clusters with an overall high support for species shown by monophyletic clustering. This effectiveness for the genus *Hoplias* is similar to that found in other fish genera [74–76].

Although the general limits of DNA Barcoding to identify species have been already discussed [77,78], the accuracy of any identification depends on several factors including the total number of sequences in the database belonging to the identified species, as well as the number of sequences of closely related taxa, and the quality of the sequences themselves. Moreover, an insufficient number of taxonomically verified entries, as well as the presence of lodged sequences with incorrect, outdated, inconsistent or unhelpful names can have serious implications for end-users of reference libraries [79]. For instance Durand et al. [80] detected inconsistencies in the labelling of sequences of Mugilidae deposited in GenBank in the course of dedicated barcoding surveys. Here, we updated the libraries to the genus *Hoplias* with more than 340 sequences. The continued updating of sequences lodged in BOLD is a crucial but rarely considered issue in the practical application of barcoding, hampering taxonomic decisions supported by these molecular data. Considering the unexpected molecular diversity within the *H*. *malabaricus* species complex revealed in this study, the DNA barcode sequence composition of the *H*. *malabaricus sensu stricto* presented here is of major significance. High-resolution studies based on DNA barcoding like the present work are intended to provide reference nucleotide-sequence databases that can be used in subsequent ecological, fisheries, food and other types of studies, particularly in groups with high species diversity and weakly defined taxa. The accumulation of undescribed species within a single supposed species of such importance as *H*. *malabaricus*, hampers attempts to created proper management and conservation strategies for this valuable, widespread and socio-economic relevant resource. The great challenge now for taxonomists is to determine if all these unnamed mitochondrial lineages can be formally described and for ecologists, geneticists and fishery scientists to relate their previous findings to this new scenario of genetic diversity within the genus *Hoplias*.

### Genetic divergence and diversity

Our molecular study of the hidden diversity of *Hoplias* covered a major part of the genus’s geographic range. DNA Barcoding revealed great genetic divergence among the nine species of *Hoplias* morphologically identified. Pereira et al. [39] performed similar studies, but covering a smaller geographic area. They used ATPase-6 and RAG2 sequences, which have different degree of nucleotide divergence than the COI gene; however they also found deep genetic divergences between populations from different coastal basins of Brazil. The unexpected large divergence among BINs of the *Hoplias* genus is not an isolated result. Genetic divergence between cryptic lineages in other Neotropical genera is also high. Melo et al. [76] showed that 43 out of 55 pairwise distances of the 11 recognized lineages for the genus *Curimatopsis* were greater than 10% (with a maximum value of 20%). Similarly, the average divergence between different clades of the small characids placed in *Astyanax* spanned from 13 to more than 21% [81]. Genetic divergence increases several folds from lower to higher taxonomic levels [82]. In fishes, average genetic distance between samples ranges from less than 1% within species to slightly more than 16% within families, but the largest genetic divergence within genera was greater than 20% [31,83]. However, most of these studies were limited to the species diversity of a regional fish fauna [24,31,71]. Only recently have studies aimed to test the diversity and power of barcoding for fish species discrimination within a genus or a family [14,76,80,81,84] and assessing the divergences among related species. A survey of several papers on Neotropical freshwater fishes indicated that congeneric COI divergence averages approximately 8% [14,23,24,31]. In *Tetragonopterus* [85] and *Neoplecostomus* [86] this value rises to 12%, but values as large as 20% were also reported [76,81,87,88] for several other genera-based approaches. Alternatively, low values of COI divergence were also reported for well-defined species in fully supported mitochondrial lineages: 1.2% in *Prochilodus* [89], 1% in *Neoplecostomus* [86] and 1.6% in *Tometes* [90]. Similarly, our results showed that genetic divergence between fully supported OTUs of *Hoplias*, ranged from slightly over 1% to more than 20%. Particularly, genetic divergence between recently described species (*H*. *mbigua*, *H*. *argentinensis* and *H*. *misionera*) ranged from 7.2 to 8.5%, closely resembling the mean congeneric distance for other Neotropical fish. These results preclude the usage of a threshold of COI divergence to postulate candidates for new cryptic species within Neotropical freshwater fishes. As shown, a range of genetic divergences (from slightly over 1% to more than 20%) has been reported between well supported MOTUs of taxonomically validated species in many genera. The ABGD, based on our data set, resulted in 26 groups perfectly matching the respective OTUs recovered by BIN analysis. Moreover, these two methodologies largely agreed with NJ and BI topologies showing well-defined branches for each BIN or Group from ABGD. Overall, 22 OTUs in *Hoplias* were supported by the three approaches and may be interpreted as “full” OTUs and 6 were only partially supported (one or two analyses). The BIN ABZ3047 was considered polyphyletic in the phylogenetic tree but was considered as a single group (Group 1) by the ABGD. However, in this group, the ABGD also included the singletons JX112679 and JX112687 (not assigned to a particular BIN), which were monophyletic with one of the three clades of ABZ3047. On the other hand, the BIN ADG3391 was not reciprocally monophyletic, being also separated into two groups (Group 7 and 8) in the ABGD. Finally, two of the “partial” OTUs were only analyzed by the BIN approach because they are private data in the BOLD System and therefore the consistency of the ABGD and phylogenetic approaches could not be tested. In most cases, there was also a clear geographic separation of the BINs and groups in ABGD in different drainage basins. Such diversification patterns can be associated with evolutionary forces promoted by ecologically driven adaptive divergence [91].

Being fully supported by three different approaches, this study detected 22 OTUs for the genus *Hoplias*. Currently, 14 valid species are known for this genus. Other hyperdiverse groups of Neotropical fishes also hosted many mitochondrial lineages supported by different approaches, although the percentage of full agreement among different approaches is rather lower than the one observed in *Hoplias*. For instance, only 41% and 50% of detected OTUs were fully supported by complementary methodologies in *Rineloricaria* [84] and *Astyanax* [81] respectively.

With the continental-scale analysis of the genus *Hoplias* conducted in this study, DNA Barcoding was able to provide private BINs for *H*. *mbigua*, *H*. *misionera*, *H*. *argentinensis*, *H*. *intermedius*, *H*. *lacerdae* and *H*. *australis*. All these BINs were further supported by the ABGD and phylogenetic analyses. Conversely, the Barcode sequence composition of *H*. *aimara* and *H*. *curupira* still needs to be solved given that different BINs with large genetic distances were discovered within these species. Moreover, one BIN of *H*. *curupira* (ADG3391) was not supported by the ABGD or phylogenetic analyses. In addition, the geographic coverage of this study, albeit extensive, was not fully comprehensive and several basins from which these species are known could not be sampled. The occurrence of more mitochondrial lineages cannot, therefore, be ruled out. These results suggest that a comprehensive taxonomic revision of *H*. *aimara* and its junior synonym *H*. *macrophtalmus* [43] should be performed to resolve the status of this taxon.

This study has revealed an unsuspected molecular diversity of 15 fully supported mitochondrial lineages that are currently “masked” within the *H*. *malabaricus* species complex. Seven OTUs within this complex were found in the La Plata River Basin, three of them represented by specimens morphologically identified as *H*. *mbigua*, *H*. *argentinensis* and *H*. *misionera*. The remaining mitochondrial lineages within *H*. *malabaricus* species complex were from elsewhere in South America, including several Amazon drainages and Northern Atlantic Rivers of Brazil. These results closely agree with previous cytogenetic studies that revealed the existence of at least eight different karyomorphs of *H*. *malabaricus*. Moreover, some of these cytotypes were found to live in sympatry without evidence of hybridisation [92–96] reinforcing the existence of different evolutionary units. Altogether, cytogenetic and molecular evidences demonstrate the existence of a large number of different evolutionary lineages within the *H*. *malabaricus* complex. Assuming that each of these evolutionary units might eventually represent a single species, the species richness within this complex would be astonishing. Therefore, taxonomical revisions and new species descriptions are imperatives. Certainly, not all the mitochondrial lineages and karyomorphs within *H*. *malabaricus* will ultimately constitute different species. In this respect, unambiguously linking each taxonomic entity with its corresponding molecular and cytotype identity in this species complex is of high importance. Three species have been recently described [45–47] and the composition of their COI sequences represent additional diagnostic characters aiding their identification. Nevertheless, they only account for three of the 15 mitochondrial lineages detected by this study for the *H*. *malabaricus* species complex. Many of these molecular lineages belong to the Amazon River Basin, in Brazil, Bolivia and Peru. Therefore, it is plausible to expect that more hidden diversity will be detected as the more remote areas of these drainages are explored.

### Biogeography of the genus *Hoplias*

Correct taxonomy and distribution data are important for conservation planning [97] and for supporting assessments made under Red List criteria [98]. Georeferenced distribution data are particularly needed to estimate the extent of occurrence, a crucial aspect for extinction risk assessments [99]. Our results provide new georeferenced data about the distribution of several species of *Hoplias*. The geographic distribution range of *Hoplias lacerdae* and *H*. *australis* were expanded with new localities in the Uruguay River Basin. Similarly, specimens of *H*. *mbigua* were collected in the Paraguay River (Argentina) and the BIN analysis suggested that this species may be also present in the Paranapanema River (Brazil), approximately 700 km north of the type locality (currently the only known). The geographic distribution of *H*. *misionera* was also greatly expanded. According to our samplings, *H*. *misionera* is distributed in the Paraguay (Argentina), Upper and Lower Paraná and Uruguay rivers. The BIN analysis suggests that this species is also present in the Amazon River and the Pilcomayo River in Bolivia. Therefore, this species, with a robust molecular identity (AAB1732, Group 17 and reciprocal monophyly), could be the most widely distributed of the genus. In contrast, our results showed that the mitochondrial lineage of *H*. *malabaricus* from the Guiana Shield, presented a restricted geographic distribution. The specimens of *H*. *malabaricus* from Suriname and French Guiana clustered in the BIN ABZ3047 and Group 1 of the ABGD, totally separated from the OTUs formed by the representatives of the La Plata River Basin. All specimens of the *H*. *malabaricus* species complex from the La Plata River Basin accordingly received different BINs and Group numbers in ABGD. Interestingly, cytogenetic evidence largely supports the geographic restriction of the mitochondrial lineage (ABZ3047 and Group 1) from Guiana Shield suggested by DNA Barcoding data. In particular, the karyomorph F from populations of Suriname is shared only with populations of Amazon and northern Atlantic drainages rivers of Brazil [100].

Disjunct distributions were observed in three of the 25 BINs. Specimens sharing the BIN AAB1732 of the recently described *H*. *misionera* were present in two distinct basin systems. The distribution of this species can be explained by recent temporary connections or river captures between the southern tributaries of the Amazon and northern tributaries of the Paraguay River. Already suggested by several authors [101–105], we provide new evidence for faunal exchanges between the Amazon Basin and the Paraguay Basin that appear to have had a semipermeable divide allowing inter-basin fish dispersal [106]. The distribution of the BIN AAZ3734 (belonging to the recently described *H*. *argentinensis*) in the lower La Plata River and the Dos Patos- Mirim Lagoon supports the previous postulated hypothesis that headwater captures have occurred between these systems. Ribeiro [107] and Albert & Reis [108] suggest that fish dispersal events between the Parana River and Eastern coastal rivers of Brazil occurred between 15 to 28 millions years ago. However, Montoya-Burgos [102] proposed a more recent fauna exchange (4.2 millions years ago). All the specimens of the *H*. *malabaricus sensu stricto* (ABZ3047) from Suriname clustered with some specimens from the Sao Francisco and Amazon rivers, which received the same BIN. Similarly, the *H*. cf. *malabaricus* from northern Atlantic drainages of Brazil (ACR9466) seem to be more intimately related to the specimens of *H*. *malabaricus sensu stricto* than to the remaining mitochondrial lineages. Several morphological and genetic studies have shown that fishes from the Guiana Region display phylogenetic positions nested within Amazonian lineages, suggesting that they originated from Amazonian ancestors [102,109–111]. The past relationships of the Amazon Basin with the Sao Francisco and northern Atlantic drainages of Brazil are not as clear.

Conversely to these widespread OTUs, most mitochondrial lineages in *H*. *malabaricus* species complex seemed to be restricted to a particular basin. In the upper Paraná River Basin several fully supported OTUs of *H*. *malabaricus* species complex (AAB1733; AAI8239; AAI8240 and ACK8876) were found to live in sympatry. The widespread AAB1732 of *H*. *misionera* and ACO5223 of *H*. *mbigua* also were found in sympatry with the formers OTUs. Similar findings for the upper Paraná River were detected in *Neoplecostomus*, where 7 species were living in sympatry [86]. In our survey, the lineage ADG3393 from Huallaga and Ucayalí rivers in Peru as well as ACK2158 from the Beni River (a tributary of the Madeira River) in Bolivia, were isolated lineages without contact to other *H*. *malabaricus* OTUs. Interestingly, the genus *Curimatopsis* also showed exclusive isolated species for the same drainages, with *C*. *macrolepis* from the Huallaga River Basin and *Curimatopsis* sp. from the Madeira River Basin [76]. Furthermore, *C*. *crypticus* were restricted to Mid Amazon- Suriname drainages, a geographic distribution observed for ABZ3047 in our study, which also was found in the Sao Francisco River Basin.

### Phylogenetic analyses

The COI sequences used in the present work gave a relatively high phylogenetic signal to noise ratio, and they seem to be well suited to detect emerging mitochondrial diversification in the genus *Hoplias*. This molecular approach agrees with the previous morphological studies, supporting the division of *H*. *malabaricus* group and *H*. *lacerdae* group as defined by Okayawa & Mattox [44]. Nevertheless, our NJ and BI trees showed with a strong support that *H*. *aimara* belongs within the *H*. *lacerdae* clade. In this respect, the diagnostic morphological characters of *H*. *aimara* would only apply at the species level. Overall, considerable effort is still required to clarify the phylogenetic relationships among the lineages and species of the genus *Hoplias*. Further, nuclear molecular markers are needed to support our phylogenetic hypothesis. Similarly, the inclusion of the remaining species of the genus as well as specimens of the species included here from geographic areas not covered in this study certainly will provide a more comprehensive understanding of the evolutionary history of the genus *Hoplias*.

Phylogenetic trees allowed us to detect some misidentifications. Several specimens from the Parapanema and Tibaji rivers (Brazil) were deposited as *H*. *intermedius* [31,73] in Genbank and BOLD System. These sequences were assigned here to two different OTUs (AAI8240 or Group 3, AAI8239 or Group 26 respectively) that belong to *H*. *malabaricus* clade. Other sequences from the Mucurí and Jaboticatubas (Sao Francisco Basin) rivers also were identified as *H*. *intermedius* [23] and assigned to OTU AAB1734 or Group 16, which belongs to *H*. *lacerdae* clade. As the type locality of *H*. *intermedius* is the Cipo River, a tributary of the Sao Francisco River (Brazil) and *H*. *intermedius* is considered a member of *H*. *lacerdae* group [44], we considered that the OTU recovered by the BIN AAB1734 and Group 16 undoubtedly represents *H*. *intermedius* and that the specimens from Parapanema and Tibaji rivers were misidentified. Some of these specimens were part of a larvae monitoring program where misidentifications are known to be common although very unfortunate. Misidentification of larvae can lead to uncertainty about the spatial distribution of a species, confusion over life history traits and population dynamics, and more problematically, disguise the collapse or recovery of populations [112].

## Supporting information

S1 Table All used sequences. Annotated list of all used sequences of *Hoplias*. BIN: Barcode Index Number. % similarity: percentage of similarity between public sequences and the nearest BOLD sequence with a known BIN. BIN Tax ID: current taxonomy.
(XLSX)

S2 Table Genetic divergence. Estimates of genetic divergence between BINs based on the Tamura-Nei model with a gamma distribution (shape parameter = 1). The final data set involved 344 nucleotide sequences with 652 base positions.
(XLS)
